# Hierarchically Porous Carbon Microspheres Coated with MnO_2_ Nanosheets as the Sulfur Host for High-Loading Lithium–Sulfur Batteries

**DOI:** 10.3390/molecules29245881

**Published:** 2024-12-13

**Authors:** Liqin Dai, Zonglin Yi, Lijing Xie, Fangyuan Su, Xiaoqian Guo, Zhenbing Wang, Jiayao Cheng, Chengmeng Chen

**Affiliations:** 1 Shanxi Key Laboratory of Carbon Materials, Institute of Coal Chemistry, Chinese Academy of Sciences, Taiyuan 030001, China; 2 CAS Key Laboratory of Carbon Materials, Institute of Coal Chemistry, Chinese Academy of Sciences, Taiyuan 030001, China

**Keywords:** lithium–sulfur batteries, porous carbon microspheres, MnO_2_ nanosheets, high sulfur loading, catalytic conversion

## Abstract

Lithium–sulfur (Li–S) batteries have emerged as a promising candidate for next-generation high-energy rechargeable lithium batteries, but their practical application is impeded by the sluggish redox kinetics and low sulfur loading. Here, we report the in situ growth of δ-MnO_2_ nanosheets onto hierarchical porous carbon microspheres (HPCs) to form an HPCs/S@MnO_2_ composite for advanced lithium–sulfur batteries. The delicately designed hybrid architecture can effectively confine LiPSs and obtain high sulfur loading up to 10 mg cm^−2^, in which the inner carbon microspheres with a large pore volume and large specific surface area can encapsulate high sulfur content, and the outer MnO_2_ nanosheets, as a catalytic layer, can improve the conversion reaction of LiPSs and suppress the shuttle effect. The thick HPCs/S@MnO_2_ electrode with 7 mg cm^−2^ sulfur loading delivers an areal capacity of 4.0 mAh cm^−2^ at 0.1 C and provides stable cycling stability with a low-capacity decay rate of 0.063 % per cycle after 200 cycles at 0.1 C. Furthermore, a Li–S pouch cell with a capacity of 2.5 A h is fabricated and demonstrates high cycling stability. This work offers a feasible method to build advanced sulfur electrodes with high areal loading and sheds light on their commercial application in high-performance Li–S batteries.

## 1. Introduction

Lithium–sulfur (Li–S) batteries have become one of the most promising candidates for high-energy rechargeable lithium batteries in the next generation due to their extremely high theoretical discharge specific capacity of 1675 mAh g^−1^, low cost, and environmentally benign nature of sulfur [1,2,3]. However, the commercialization of Li–S batteries is severely hindered by several challenges, including the low conductivity of S/Li_2_S, the shuttle effect of dissolved polysulfides, and low sulfur loading [4,5,6]. These effects lead to the low utilization of active sulfur, a short cycle life, and poor rate performance [7,8,9].

Extensive efforts have been devoted to solving these problems. The widely used strategy is to encapsulate sulfur into conductive, large-specific-surface-area porous carbonaceous materials (e.g., hierarchical porous carbon [10,11], carbon nanowires/nanotubes [12,13], and graphene [14]) to inhibit the shuttle effect of lithium polysulfides (LiPSs). However, the capacity still decays rapidly under long cycles, owing to the weak interactions between non-polar carbon materials and polar polysulfide species [15,16]. Recently, polar materials, including metal oxides (MnO_2_, TiO_2_, MoO_2_) [17,18,19,20] and metal sulfides (MoS_2_, CoS_2_, FeS_2_) [21,22,23], have been employed to effectively suppress the shuttle effect and improve the long-time cycling stability by creating strong chemical bonds with LiPSs. Among the polar materials, δ-MnO_2_ is regarded as a remarkable chemical confinement of LiPSs, which could effectively inhibit shuttle effects and accelerate the catalytic conversion of intercepted polysulfides into insoluble Li_2_S [24,25,26,27]. Despite achieving outstanding advances, their relatively low electrical conductivity inevitably increases the impedance and reduces the rate capability. Moreover, the low sulfur loading for polar materials severely limits the commercial application of Li–S batteries. The combination of highly conductive carbon materials and polar materials as sulfur host materials is a rational solution. Very recently, the carbon/MnO_2_ hybrid sulfur host has been reported to exhibit a synergistic effect in efficiently encapsulating sulfur for long cycles. For example, Li et al. reported hollow carbon nanofibers (HCFs) encapsulated with MnO_2_ nanosheets as the sulfur host, which displayed a reversible capacity of 662 mAh g^−1^ after 300 cycles at 0.5 C [28]. Chen et al. reported hollow nitrogen-doped carbon filled with MnO_2_ nanosheets as a sulfur host, exhibiting a high reversible capacity of 1023 mAh g^−1^ after 200 cycles at 0.2 C [29].

However, most of these reported composite cathodes deliver areal sulfur loading below 2 mg cm^−2^, which is far from the practically applied requirements for high-energy-density lithium–sulfur batteries. It is essential to increase the areal sulfur loading of cathodes to achieve a high energy density of Li–S batteries. The practically accepted areal sulfur loading level is 3–5 mg cm^−2^ or higher [30,31]. Eroglu et al. described more challenging cell parameters for Li–S batteries applied in electric vehicles, of which the sulfur loading should be higher than 7.0 mg cm^−2^ [32]. Unlike traditional lithium-ion batteries, the electrochemical performances of Li–S batteries are quite sensitive to the areal sulfur loading, and many new problems arise under high sulfur loading. The thick electrodes usually suffer from more severe polysulfide shuttle effects and fast capacity degradation because of their poor electrolyte wetting, electrode cracking, and peeling-off problems [33,34]. The main challenge is to regulate the particle design, electrode architecture, and interfacial properties of cathode hosts to increase the porosity of thick electrodes for better electrolyte wetting and fast Li-ion reaction kinetics with active sulfur [30]. Some scholars have found that the thick electrode prepared with microsized secondary particles composed of nanoparticles is beneficial to the electrolyte’s uptake [34,35]. Moreover, the rational design of core/shell microstructured cathodes can prevent the loss of sulfur and stabilize its structural integrity, which was proposed to facilitate high-sulfur-loading Li–S batteries [18]. It should be noted that the design of internal core particles and properties of shell materials play a vital role in structural integrity, mechanical robustness, and shuttle effect inhibition. Moreover, from an industrial perspective, the synthetic method of cathodes must be easy, cost-effective, and beneficial for preparing high-mass-loading electrodes by slurry coating for the practical application of Li–S batteries. It is urgently needed to design cathodes with a reasonable structure to deliver a high areal capacity and long cycling life for superior Li–S batteries.

Herein, we present a rational design strategy to achieve high-sulfur-loading cathodes and effectively confine LiPSs by the in situ growth of δ-MnO_2_ nanosheets onto hierarchical porous carbon microspheres (HPCs) as the sulfur host. The unique structure has the following advantages. Firstly, the inner three-dimensional (3D) HPCs can not only offer fast channels to promote the transfer of electrons and Li-ions for maximum sulfur utilization but also physically entrap polysulfides due to their large specific surface area and high pore volume. In addition, the outer MnO_2_ nanosheet shell can suppress the shuttle effect via the chemical adsorption of LiPSs and fast conversion of LiPSs to insoluble Li_2_S. Furthermore, the microsphere structured cathodes can afford high-mass sulfur loading and improve the electrolyte wettability of the thick electrode to enhance the reaction kinetics. Remarkably, through structural and chemical encapsulations of LiPSs, the HPCs/S@MnO_2_ composite cathode exhibits excellent electrochemical performance, and the electrode with 4–10 mg cm^−2^ high sulfur loading can be achieved. The composite exhibits enhanced rate capability up to 5 C at sulfur loading of 4.0 mg cm^−2^. When the sulfur loading increases to 7.0 mg cm^−2^, the areal capacity attains 4.0 mA h cm^−2^ at 0.1C and keeps a low-capacity fading rate of 0.063 % per cycle after 200 cycles, approaching the requirements of practical application. The cathode exhibits high specific capacity of 1077.2 mAh g^−1^ at 0.05 C even at extra-high sulfur loading of 10.0 mg cm^−2^. The cathode can be easily scaled up and a pouch type Li–S battery with a capacity of 2.5 Ah was assembled.

## 2. Results

### 2.1. Microstructure and Composition

**Figure 1:**
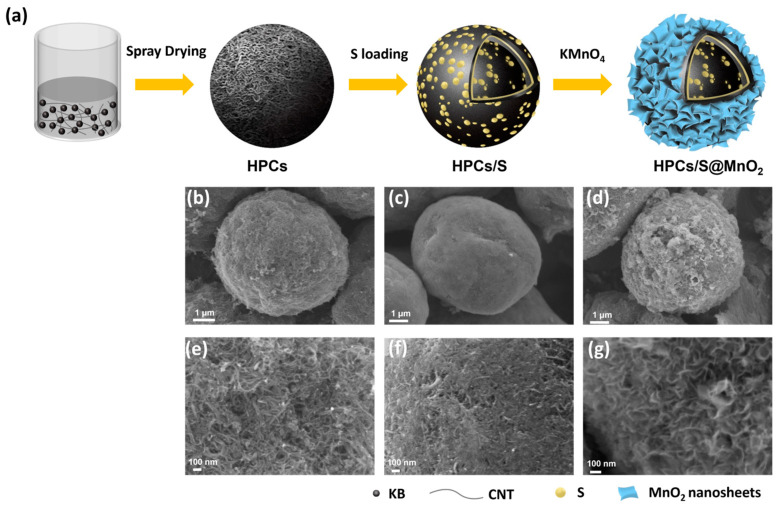
(**a**) Schematic illustration of the synthesis of HPCs/S@MnO_2_ composite and the corresponding SEM images of (**b**,**e**) HPCs, (**c**,**f**) HPCs/S, and (**d**,**g**) HPCs/S@MnO_2_ composites.

The synthesis process of the HPCs/S@MnO_2_ composite is schematically illustrated in Figure 1a. Firstly, the HPCs are synthesized by spray-drying mixtures of Ketjenblack (KB) and multiwalled carbon nanotubes (MWCNTs), taking advantage of the large specific surface area of KB and woven structure of MWCNTs. The SEM image of HPCs (Figure 1b) exhibits uniform spherical microparticles with an average size of 10 μm. At higher magnifications (Figure 1e), the HPCs clearly demonstrate an intertwined structure composed of KB nanoparticles and MWCNTs, which is proved by the hierarchical porous structure. Subsequently, sublimed sulfur is impregnated into the as-prepared HPCs by the melt-diffusion method to obtain HPCs/S composite. After sulfur impregnation, the surface of HPCs/S becomes relatively dense and smooth (Figure 1c,f), indicating that sulfur has successfully infiltrated into the hierarchically porous carbon spheres. Finally, the MnO_2_ nanosheets are generated on the surface of HPCs/S by in situ redox reaction of KMnO_4_ with S and C in HPCs/S, and the redox reaction can be described by Equations (1) and (2) [18,36]. The SEM image of HPCs/S@MnO_2_ (Figure 1d) displays a rough surface and the nanosheet-like MnO_2_ layer is generated (Figure 1g).

6KMnO_4_ + 3S + H_2_O = K_2_SO_4_ + 6MnO_2_ + KOH + K_3_H(SO_4_)_2_ (1)

4KMnO_4_ + 3C + H_2_O = K_2_CO_3_ + 4MnO_2_ + 2KHCO_3_ (2)

**Figure 2:**
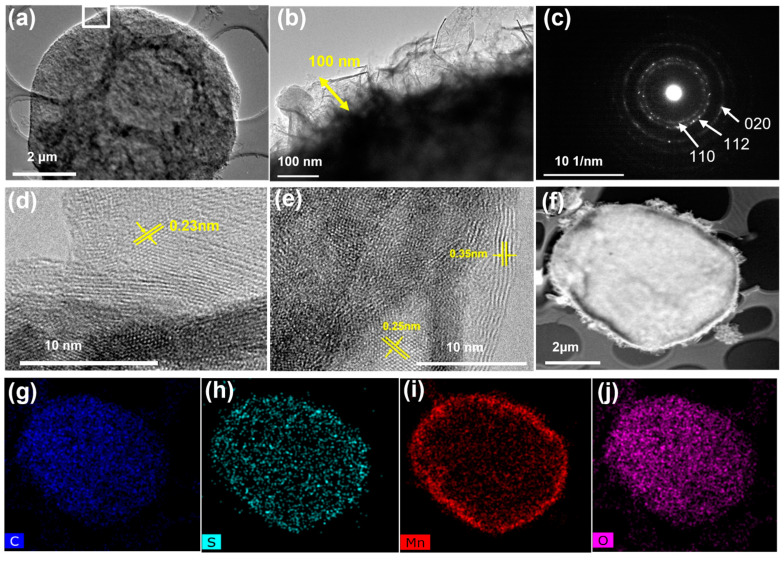
(**a**,**b**) TEM images of HPCs/S@MnO_2_ at different magnifications. (**c**) SAED pattern. (**d**,**e**) HRTEM images of the MnO_2_ nanosheets. (**f**) HAADF–STEM image of HPCs/S@MnO_2_ and the corresponding EDS mapping of C (**g**), S (**h**), Mn (**i**), and O (**j**) elements in the HPCs/S@MnO_2._

The TEM images (Figure 2a,b) verify that the MnO_2_ nanosheets are uniformly coated on the surface of the HPCs/S microspheres with a thickness of approximately 100 nm. The corresponding SAED of HPCs/S@MnO_2_ in Figure 2c confirms the good crystallinity of δ-MnO_2_ [29]. As shown in the high-resolution TEM (HRTEM) images of MnO_2_ nanosheets (Figure 2d,e), the lattice spacings of 0.35, 0.25, and 0.23 nm are assigned to the (002), (110), and (111) planes of birnessite δ-MnO_2_ phase, respectively. The corresponding EDX elemental mapping (Figure 2f–j) reveals the uniform distribution of the C, O, S, and obvious Mn surrounding the outer layer of the microspheres. This result indicates that sulfur is evenly encapsulated in the carbon microspheres and MnO_2_ nanosheets are successfully generated on the outer surface layer of HPCs/S microspheres. The MnO_2_ nanosheet layer and the inner hierarchically porous carbon spheres provide double-layer protection to immobilize sulfur and effectively suppress the shuttle effects.

**Figure 3:**
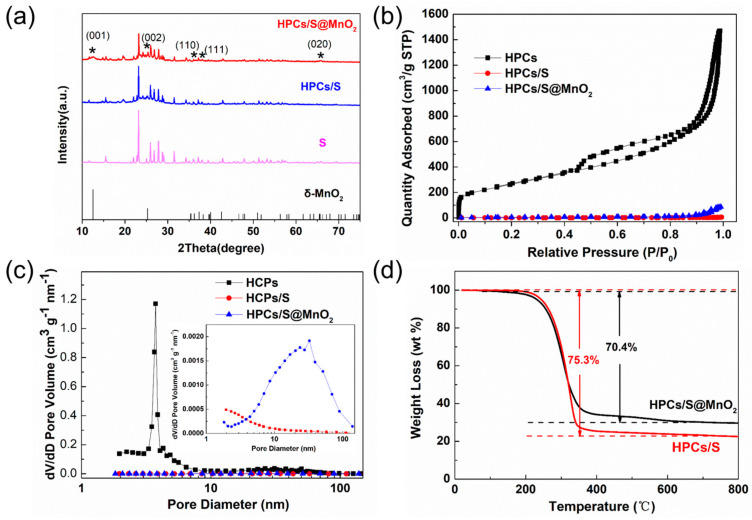
(**a**) XRD patterns of δ-MnO_2_, sublimed sulfur, HPCs/S, and HPCs/S@MnO_2_ (* denotes the characteristic peak of δ-MnO_2_.) (**b**) The N_2_ adsorption–desorption isotherms of HPCs, HPCs/S, and HPCs/S@MnO_2_. (**c**) The corresponding pore size distributions (inset: the magnified pore size distribution curve of the HPCs/S@MnO_2_ (blue line) and HPCs/S (red line)) and (**d**) the TG curves of HPCs/S and HPCs/S@MnO_2_.

The XRD measurement is carried out to identify the crystalline structure of these samples. As shown in Figure 3a, the obvious diffraction peaks of HPCs/S and HPCs/S@ MnO_2_ could be indexed to the orthorhombic structure of sulfur in the two composites. The diffraction peaks of HPCSs/S@MnO_2_ at 12.5°, 25.2°, 36.2°, 39.6°, and 65.6° are assigned to the (001), (002), (110), (111), and (020) planes of the monoclinic birnessite structure (δ-MnO_2_, JCPDS 80-1098), respectively, which is in agreement with the HRTEM results of HPCs/S@MnO_2_. As illustrated in the Raman spectrum (Figure S1), the typical peaks of HPCs/S@ MnO_2_ located at 647 cm^−1^ are related to the Mn–O lattice vibrations, further confirming the existence of δ-MnO_2_ [37]. The N_2_ adsorption/desorption isotherms of HPCs with type IV isotherm (Figure 3b) indicate a mesopore-rich carbon structure, which takes advantage of the rich small mesopores of KB and large mesopores/macropores of MWCNTs (Figure S2). The pore size of HPCs is centered within the ranges of 3.0–4.5 nm and 25–50 nm (Figure 3c), verifying the hierarchical porous structure of HPCs. The rich small mesopores can effectively confine sulfur, while the large mesopores can act as channels for fast transmission of Li-ions [38,39]. The HPCs possess a large specific surface area (957.8 m^2^ g^−1^), large pore volume (2.35 cm^3^ g^−1^) (Table S1), and hierarchical pore size distribution, which makes them ideal carbon hosts for high sulfur loading. After the infiltration of sulfur, the specific surface area and the pore volume decrease to 2.4 m^2^ g^−1^ and 0.007 cm^3^ g^−1^, implying that the sulfur has successfully infiltrated into the hierarchical porous carbon spheres. However, the values of HPCs/S@MnO_2_ slightly increase to 16.8 m^2^ g^−1^ and 0.128 cm^3^ g^−1^, due to the growth of MnO_2_ nanosheets. The pore size distribution (Figure 3c) displays that new mesopores and macropores with diameters ranging from 10 nm to 100 nm are formed for HPCs/S@MnO_2_ samples. Combined with the SEM images of HPCs/S@MnO_2_ samples (Figure 1g), it can be concluded that the MnO_2_ nanosheets are porous and the stacking of MnO_2_ nanosheets leads to the formation of mesopores and macropores. The MnO_2_ nanosheets with few mesopores could accelerate the infiltration of electrolyte into the electrode, especially for the electrode with high areal sulfur loading [40]. The TGA result (Figure 3d) shows that HPCs/S possesses a sulfur content of 75.3 wt%. After the growth of MnO_2_ nanosheets, the sulfur loading decreases to 70.4 wt% for HPCs/S@MnO_2_, due to the redox reaction of sulfur with KMnO_4_ solution. The MnO_2_ content of HPCs/S@MnO_2_ is about 14.0 %, which can be confirmed by the content of 14.5 wt% from TGA studies in air atmosphere (Figure S3) and Mn content of 14.0 % measured by ICP-MS (Table S2).

**Figure 4:**
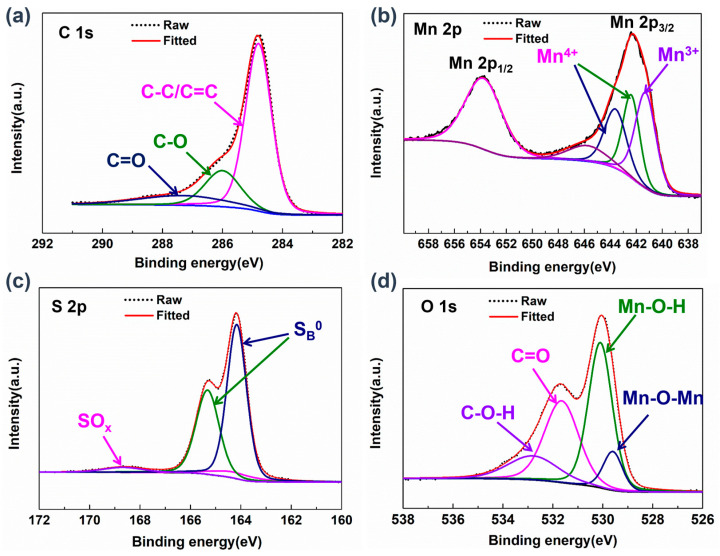
XPS spectra of HPCs/S@MnO_2_. (**a**) C 1s, (**b**) Mn 2p, (**c**) S 2p, (**d**) O 1s.

The surface chemical states of HPCs/S@MnO_2_ are characterized by X-ray photoelectron spectroscopy (XPS). As shown in Figure S4, the survey spectra of HPCs/S@MnO_2_ confirm the existence of C, O, S, and Mn elements. The high-resolution C1s spectra (Figure 4a) display three fitted peaks centered at 284.8, 286.0, and 288.5 eV corresponding to C–C/C=C, C–O, and C=O groups, respectively [41]. The spectrum of Mn 2p displays two characteristic peaks at binding energies of 653.8 and 642.3 eV in Figure 4b, which can be attributed to the Mn 2p_1/2_ and Mn 2p_3/2_, respectively. Additionally, for the Mn 2p_3/2_ spectrum, these two characteristic peaks at 642.3 and 643.6 eV are related to chemical states of Mn^4+^, while the peak at 641.4 eV is attributed to Mn^3+^ [25,42,43]. The S 2p spectrum in Figure 4c possesses one doublet at 164.1 and 165.15 eV corresponding to the elemental sulfur (S_B_^0^), and the other peak at 169.1 eV is attributed to SO_x_ species due to covalent bonds between S and MnO_2_ [44]. The spectrum of O 1s (Figure 4d) can be deconvoluted into four peaks with binding energies of 529.6, 530.1, 531.6, and 532.8 eV, which can be indexed to Mn–O–Mn, Mn–O–H, C=O, and C–O–H bonds, respectively [45,46]. The above XPS results strongly verify the coexistence of C, S, and MnO_2_ in HPCs/S@MnO_2_ and further confirmed the formation of MnO_2_ nanosheets on HPCs/S microspheres.

### 2.2. Electrochemical Performance

Electrochemical performance of the HPCs/S@MnO_2_ and HPCs/S electrodes is evaluated with coin cells (CR 2032). The cyclic voltammetric (CV) curves of HPCs/S@MnO_2_ and HPCs/S are made at a scan rate of 0.1 mV s^−1^. As shown in Figure S5, two cathodic peaks are observed at ~2.35 V and ~2.04 V, which are assigned to the reduction of S_8_ to long-chain polysulfides (Li_2_S_n_, 4 ≤ *n* ≤ 8) and further conversion to Li_2_S_2_/Li_2_S, respectively [47,48]. The anodic peak corresponds to the reverse oxidation from Li_2_S_2_/Li_2_S to S_8_ [49]. The HPCs/S@MnO_2_ electrode exhibits a higher current density and lower polarization (ΔV = 0.60 V → 0.57 V) compared to HPCs/S, demonstrating that δ-MnO_2_ nanosheets can accelerate the polysulfide redox reaction kinetics.

**Figure 5:**
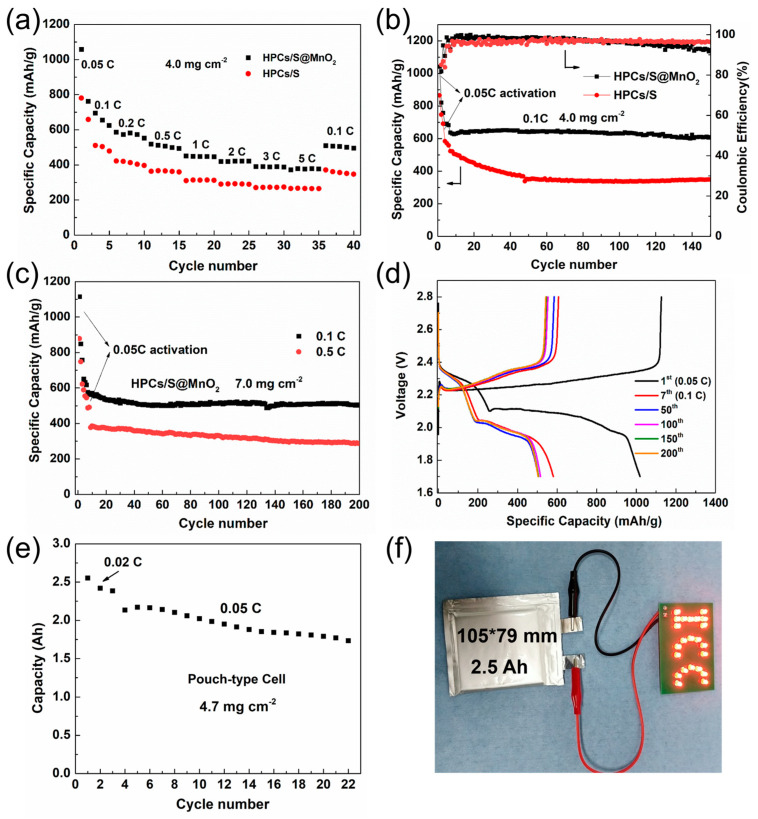
(**a**) Rate performances of HPCs/S and HPCs/S@MnO_2_ electrodes. (**b**) Cycling performances of HPCs/S and HPCs/S@MnO_2_ electrodes with mass loading of 4.0 mg cm^−2^ at 0.1 C. (**c**) Cycling performance of the HPCs/S@MnO_2_ electrodes with mass loading of 7.0 mg cm^−2^ at 0.1 C and 0.5 C. (**d**) Charging/discharging curves of the HPCs/S@MnO_2_ with mass loading of 7.0 mg cm^−2^ at 0.1 C. (**e**) Cycling performance of pouch type cell. (**f**) Digital graphical photo of pouch cell in working state.

High areal sulfur loading can significantly increase the energy density of Li–S batteries and promote their practical applications. However, electrodes with high areal sulfur loading usually suffer from fast capacity decay and low sulfur utilization, due to the poor electrolyte immersion of the thick electrodes and severe polysulfide shuttle effects. Therefore, we prepared the HPCs/S@MnO_2_ electrode with mass loading of 4, 7, 10 mg cm^−2^ and further studied the electrochemical behavior to explore its potential for practical applications. To evaluate the performance of thick electrodes under high current density, the electrochemical performance of the HPCs/S@MnO_2_ and HPCs/S electrode with high mass loading is explored. The rate performance of the HPCs/S@MnO_2_ and HPCs/S electrode at a mass loading of 4.0 mg cm^−2^ is tested at different current densities ranging from 0.05 C to 5 C. As depicted in Figure 5a, the HPCs/S@MnO_2_ delivers a specific capacity of 1057.3 mAh g^−1^ at 0.05 C. When increasing the current rate to 1, 3, and 5 C, it delivers reversible capacities of 449.8, 390.3, and 378.5 mAh g^−1^, respectively. In sharp contrast, the discharge capacity of the HPCs/S only reaches 780.9 mAh g^−1^ at 0.05 C and decays rapidly with increasing current density. The cycling performance of the two composites with areal sulfur loading of 4.0 mg cm^−2^ is presented in Figure 5b, which was first activated at 0.05 C for six cycles, followed by 0.1 C in the subsequent cycles. The HPCs/S@MnO_2_ composite shows higher capacity and better cycling stability than HPCs/S at 0.1C. After pre-activation at 0.05 C for six cycles, the initial discharge capacity of the HPCs/S@MnO_2_ composite reaches 636.5 mAh g^−1^ at 0.1C, and the capacity retention is as high as 95.5% after 150 cycles, with a very low-capacity decay rate of 0.03 % per cycle. Meanwhile, the HPCs/S cathode shows an inferior discharge capacity and retains only 66.7 % of the initial capacity after 150 cycles. Furthermore, compared with other reported typical sulfur hosts, the HPCs/S@MnO_2_ also exhibits good cycle stability at high mass loading (Table S3) [17,20,26,50,51,52,53,54,55,56]. The enhanced rate and cycling performances of the HPCs/S@MnO_2_ cathode clearly illustrate the function of MnO_2_ nanosheets in accelerating the redox kinetics of polysulfides and improving the utilization of the active materials.

It is noted that the designed HPCs/S@MnO_2_ electrode still delivers stable and high specific capacities at higher mass loading of 7 mg cm^−2^. As depicted in Figure 5c, the cell is first cycled at 0.05 C for six activation cycles and then charged and discharged at current density of 0.1 C and 0.5 C, respectively. The HPCs/S@MnO_2_ composite shows an initial discharge capacity of 574.3 mAh g^−1^ (4.0 mAh cm^−2^) at 0.1 C and maintains a low-capacity decay rate of 0.063 % per cycle after 200 cycles. When cycled at a high rate of 0.5 C, the HPCs/S@MnO_2_ composite also delivers excellent electrochemical stability with a capacity fade rate of 0.118%. The corresponding charge/discharge voltage profiles of HPCs/S@MnO_2_ (Figure 5d) at 0.1 C demonstrate two distinct voltage plateaus at ~2.35 V and ~2.04 V during the discharge process, which is consistent with the two reduction peaks in CV curves. During activation at 0.05 C, the HPCs/S@MnO_2_ exhibits a high specific capacity of 1114.3 mAh g^−1^ in the first cycle. When cycled at 0.1C from the 7th cycle to the 200th cycle, the discharge voltage plateau drops slowly, suggesting a more stabilized electrochemical performance of HPCs/S@MnO_2_. Excitingly, when the mass loading reaches 10 mg cm^−2^, the HPCs/S@MnO_2_ cathode delivers an initial specific capacity of 1110.0 mAh g^−1^, corresponding to an initial area capacity of 11.1 mAh cm^−2^ at 0.05 C (Figure S6). The discharge/charge voltage profiles show two typical voltage plateaus, further implying its high utilization of active sulfur. However, the capacity decays significantly when increasing the current density to 0.1 C. The high sulfur loading of 10 mg cm^−2^ is performed to explore the loading limits of HPCs/S@MnO_2_. By optimizing the type of binder and coating formula, the performance of 10 mg cm^−2^ may be fully realized under high current density, and we will explore it in further works. The remarkable performances of the HPCs/S@MnO_2_ cathode with high sulfur loading can be attributed to the porous carbon microspheres and strong chemical catalytic effect of MnO_2_ nanosheets, which provide fast electronic/ionic transport and promote conversion of LiPSs to Li_2_S_2_/Li_2_S.

To further estimate its practical application in Li–S batteries, an up-scaled HPCs/S@MnO_2_ (~100 g) is prepared and pouch cells with a capacity of 2.5 Ah are fabricated. The electrodes with mass loading of 4.7mg cm^−2^ and a low electrolyte-to-sulfur ratio of 3.0 μL mg^−1^, are applied in our Li–S cell, and the pouch cell exhibits specific energy densities of 270.6 Wh kg^−1^. High initial specific discharge capacity of 1118.1 mAh g^−1^ is obtained, and the discharge curves deliver two typical plateaus (Figure S7). As shown in Figure 5e, the cycling performance at 0.05 C is relatively good with capacity retention of 81.3% after 22 cycles. In addition, 23 LEDs could be easily lit by our Li–S pouch cell, which certifies the practical potential of the HPCs/S@MnO_2_ cathode for commercialization (Figure 5f).

**Figure 6:**
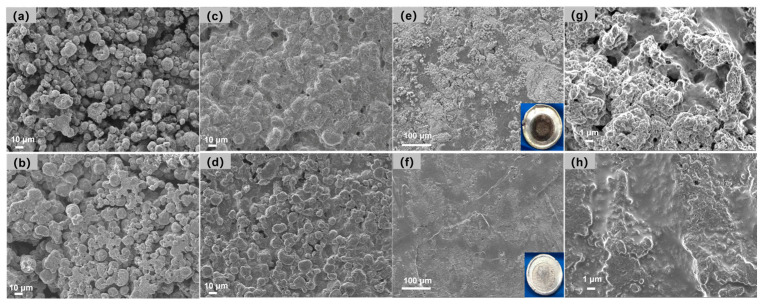
SEM images of original electrode of (**a**) HPCs/S, (**b**) HPCs/S@MnO_2_ and cycled electrode of (**c**) HPCs/S, (**d**) HPCs/S@MnO_2_; the SEM images and higher magnification of cycled lithium anode (**e**,**g**) HPCs/S cell and (**f**,**h**) HPCs/S@MnO_2_ cell (inset: photograph of the corresponding cycled lithium anode).

To further explore the structure–performance relationship of the high-loading electrode, the morphology of sulfur electrodes and lithium anodes after cycling are observed. After 150 cycles at 0.1 C, the Li–S cells of HPCs/S and HPCs/S@MnO_2_ with areal sulfur loading of 4.0 mg cm^−2^ are disassembled and characterized. As shown in Figure 6a,b, both original electrodes exhibit a loose structure composed of spherical particles. However, profound changes are observed in the cycled HPCs/S electrode. The surface of the cycled HPCs/S electrode (Figure 6c) becomes dense and is completely covered by LiPSs precipitates. Meanwhile, the cycled HPCs/S@MnO_2_ electrode exhibits slight aggregation of LiPSs and maintains its porous microsphere structure (Figure 6d), which could provide sufficient channels for Li-ion transportation and ensure excellent electrochemical performance. Furthermore, the comparison of the surface morphology of cycled lithium anodes facing the cathode side also verifies the merits of the HPCs/S@MnO_2_ cathode in the high-sulfur-loading cell. A black passivation film is revealed on the Li anode surface of the HPCs/S cell (Figure 6e), indicating a severe anodic corrosion. The morphology via SEM clearly shows a highly powdered and porous structure with extensive dendritic Li growth (Figure 6g), which is derived from severe side-reactions between the electrolyte and Li anode. By contrast, the lithium surface of the HPCs/S@MnO_2_ cell is relatively flat and less dense Li deposition is formed (Figure 6f,h). Owing to the fast catalytic conversion ability of LiPSs provided by MnO_2_, the LiPSs’ dissolution and migration to the surface of Li anodes are greatly inhibited, resulting in slight anodic corrosion and good cycling stability at high sulfur loading.

**Figure 7:**
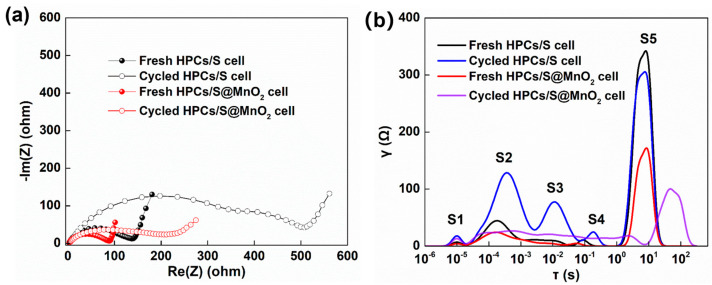
EIS results of fresh and cycled HPCs/S and HPCs/S@MnO_2_ cell (**a**) and DRT calculated from EIS measurements (**b**).

The good electrochemical kinetics of HPCs/S@MnO_2_ is further evaluated by electrochemical impedance spectra (EIS). As shown in Figure 7a, the Nyquist plots of the HPCs/S cell before and after cycling have a huge difference. The high-frequency semicircle of the HPCs/S electrode, corresponding to charge transfer resistance (Rct), increases remarkably after 150 cycles. In contrast, the high-frequency semicircle of HPCs/S@MnO_2_ increases slightly, implying the faster charge transfer kinetics of HPCs/S@MnO_2_. The distribution of relaxation times (DRT) can quantify the kinetics of the major electrochemistry process in the timescale, of which the time constant (τ) represents each electrochemical process and the integral area is characteristic of the resistance of the process [57]. The DRT plot derived from EIS data is revealed in Figure 7b. The DRT spectra consist of five characteristic peaks in the relaxation time range τ = ~10^−5^ s, 10^−3^~3 × 10^−4^, 10^−2^~10^−3^, 10^−1^~10^0^ s, 10^0^~10^2^ s, corresponding to ohmic resistances resulting from ionic resistance and electronic resistance of the carbon electrodes (S1), Li-ion migration of the lithium anode and the polarization of the positive electrode (S2), charge transfer and formation of solid products on the positive electrode (S3), diffusion of the LiPSs at the positive electrode (S4), and bulk Li-ion diffusion (S5), respectively [58,59,60]. The resistance of all peaks in HPCs/S is significantly higher than that of HPCs/S@MnO_2_, especially for the cycled cell, indicating that all electrochemical processes are hindered. As shown in Figure 6, the very dense isolating layers on the surface of the cathode and severe anodic corrosion are the main reasons for the increased resistance of the cycled HPCs/S cell. The DRT results indicate that the MnO_2_ nanosheets can greatly inhibit the polarization of the lithium anode and positive electrode, accelerate the electron transfer, and promote the movement of ions to participate in reactions, which greatly suppress the formation of the insulating Li_2_S/Li_2_S_2_ layer on the electrode surface during the cycling process.

**Figure 8:**
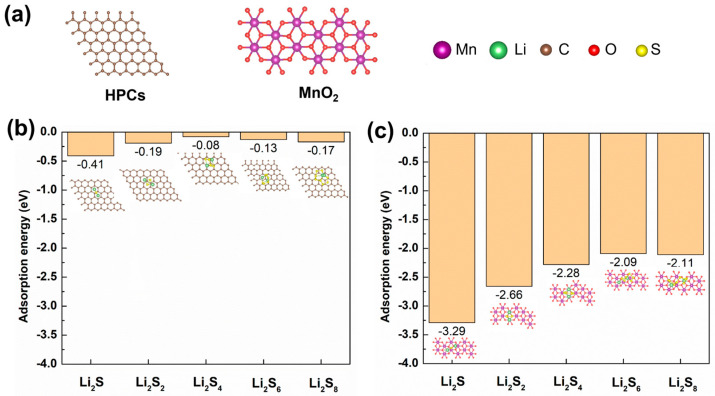
(**a**) Theoretical structures of HPCs and MnO_2_. DFT calculation optimized geometric configurations and binding energies for the adsorption of polysulfides onto the HPCs (**b**) and MnO_2_ (**c**).

Density functional theory (DFT) calculations (Figure 8) are carried out to study the interactions of lithium polysulfide species on the carbon surface and MnO_2_ nanosheets (100) surface. The chemical bonds of Li–O are observed, and the corresponding binding energies of Li_x_S_n_ species with HPCs and MnO_2_ nanosheets are plotted in Figure 8b,c. The binding energy between MnO_2_ and Li_2_S_x_ is more negative than that between HPCs and Li_2_S_x_, indicating stronger adsorption of the MnO_2_ for LiPSs. To further verify the adsorption ability of MnO_2_ nanosheets for the polysulfides, 5 mg of HPCs and HPCs@MnO_2_ are separately added to 3 mL of 0.02 M Li_2_S_4_ solution and the change in color is observed. As shown in Figure S8, the Li_2_S_4_ solution becomes nearly colorless after 1 h after adding HPCs@MnO_2_, while the color of Li_2_S_4_ solution containing HPCs still remains light yellow, illustrating outstanding adsorption ability of HPCs@MnO_2_ towards LiPSs.

**Scheme 1:**
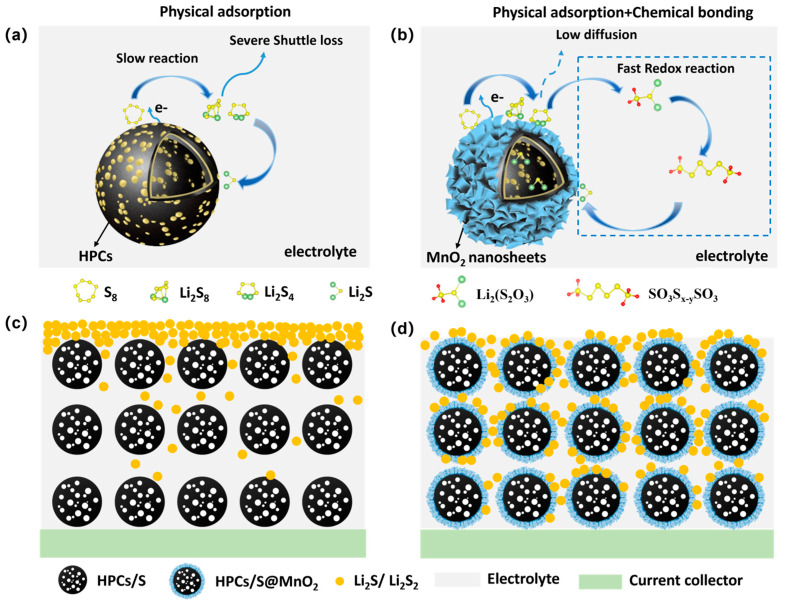
Schematic illustration of polysulfide adsorption via HPCs/S (**a**) and HPCs/S@MnO_2_ (**b**); Schematic diagrams of morphologies of HPCs/S (**c**) and HPCs/S@MnO_2_ cathode (**d**) after discharging.

The reaction mechanism of polysulfides with HPCs/S and HPCs/S@MnO_2_ during the discharge process is revealed in Scheme 1. The 3D hierarchically porous carbon can promote the fast transfer of electrons/Li-ions and physically adsorb polysulfides, acting as the first barrier for the shuttling of polysulfides. However, the physical adsorption is weak and cannot strongly confine the polar polysulfides, which leads to severe shuttle loss of polysulfides and capacity fading, as shown in Scheme 1a. Considering these issues, MnO_2_ nanosheets, rationally designed as another barrier, can provide strong chemical adsorption for the LiPSs. This accelerates the reduction of polysulfides by catalytic conversion, and polysulfide diffusion is greatly suppressed (Scheme 1b). Therefore, the HPCs/S@MnO_2_ exhibits fascinating advantages of physical adsorption and chemical bonding for polysulfides. The mechanism of redox interaction between LiPSs and MnO_2_ nanosheets is illustrated in Scheme 1b. Firstly, the soluble LiPSs are chemically adsorbed onto the MnO_2_ nanosheets by strong S–O interaction between the S of polysulfides and O of MnO_2_, and then are in situ oxidized to form an intermediate insoluble lithium thiosulfate (Li_2_S_2_O_3_) (Equation (3)). Subsequently, the thiosulfates further adsorb newly formed polysulfides to form polythionate complexes [SO_3_S_x−y_SO_3_]^2−^ and insoluble short-chain polysulfides S_y_^2−^ (y ≤ 2) by a disproportion reaction (Equation (4)). The polythionate complex [SO_3_S_x−y_SO_3_]^2−^ acts as an important mediator to accelerate the conversion of long-chain polysulfides to short-chain polysulfides and effectively suppress the polysulfides’ dissolution, resulting in the outstanding performance of HPCs/S@MnO_2_ [43,61].

5[MnO_6_] + Li_2_S_4_ → 4[MnO_6_] + Mn(S_2_O_3_) + Li_2_(S_2_O_3_) (3)

[S_2_O_3_]^2−^ + S_x_^2−^ → [SO_3_S_x−y_SO_3_]^2−^ + Sy^2−^ (x ≥ 4, y < 3) (4)

The mechanism of two cathodes based on thick electrodes during the discharging process is illustrated in Scheme 1c,d. The microsized spherical particles are designed to form a high-loading electrode, which can facilitate Li-ion transportation and electrolyte infiltration in this work. However, due to the weak adsorption of sulfur species for HPCs/S, severe dissolution and redistribution of unconfined polysulfides occur during cycling. The discharge products of Li_2_S_2_/Li_2_S preferentially deposit on the cathode surface and block the Li-ion transportation, ultimately resulting in the loss of active sulfur and rapid capacity decay for HPCs/S composites. As for the HPCs/S@MnO_2_ cathode, due to the catalytic effect of MnO_2_ nanosheets, the conversion reaction from LiPSs to Li_2_S_2_/Li_2_S during the discharge process is accelerated, and the redistribution of free polysulfides is greatly inhibited. Furthermore, its relatively rich mesopores and large pore volume from MnO_2_ nanosheets can provide channels and space for polysulfides to transport, and Li_2_S_2_/Li_2_S are deposited from the surface towards the interior depths of the electrode. Because of the high conductivity of porous carbon spheres, the short-chain polysulfides obtained from the catalytic conversions can deposit on HPCs, thereby avoiding passivation of the catalytic sites. Finally, Li_2_S_2_/Li_2_S are uniformly deposited on carbon microspheres, which can form an interconnected porous structure to ensure electrolyte infiltration and Li-ion transport. Overall, the unique design of HPCs/S@MnO_2_ significantly suppresses the polysulfide shuttle effects and enables the thick electrode to maintain its porous structure during the cycling process, eventually facilitating the utilization of active sulfur and ensuring excellent capacity retention.

## 3. Materials and Methods

### 3.1. Synthesis of HPCs

First, 2 kg of multiwalled carbon nanotube (MWCNT) solution (Jiangsu Cnano Technology Co., Ltd., Zhenjiang, China, LB217-54, 5 wt% of MWCNTs), 100 g of Ketjenblack (KB) (Lion Specialty Chemicals Co., Ltd., Tokyo, Japan, EC-600JD), and 3 L of deionized water were premixed using a planetary stirring machine (HY-DLH3L, Hongyun Mixing Equipment Co., Ltd., Guangzhou, China) for 2 h, then ultrasonically stirred for 1 h, and finally a homogeneously dispersed CNT/KB slurry was obtained. The CNT/KB slurry was spray-dried to obtain HPCs at a drying temperature of 120 °C, a pressure of 0.2 MPa, and a flow rate of 1 L/h (YC-015, Shanghai Pilotech Instruments and equipment Co., Ltd., Shanghai, China).

### 3.2. Synthesis of the HPCs/S Composite

The HPCs/S composite was synthesized by a melt–diffusion approach. The HPCs and sublimed sulfur were thoroughly mixed with a weight ratio of 1:3 by ball-milling for 3 h and then heated at 155 °C in a Teflon-lined stainless steel autoclave (KLJX-8A, Yantai Keli Chemical Equipment Co., Ltd., Yantai, China) filled with argon gas for 8 h to infiltrate the sulfur into the HPCs.

### 3.3. Synthesis of the HPCs/S@MnO_2_ Composite

First, 50 g of as-prepared HPCs/S was dispersed in 3 L of deionized water containing 2.5 g of PVP and stirred for 2 h at room temperature. Then, 16 g of KMnO_4_ was dissolved in 1L deionized water and then added dropwise to the above HPCs/S suspension by continuous stirring for 12 h at 60 °C. The product was collected by centrifugation and washed several times with deionized water. The final product was dried at 60 °C for 12 h.

### 3.4. Characterizations

The microcrystalline structures were characterized by the X-ray diffraction patterns (XRD, D8 Advance, Bruker, Berlin, Germany) with Cu Ka radiation (λ = 1.54 Å) and the Raman spectrum (Horiba HR800, Horiba, Kyoto, Japan) with a laser wavelength of 532 nm. The Barrett–Joyner–Halenda (BJH) pore-size distribution and Brunauer–Emmett–Teller (BET) surface area were measured via a nitrogen adsorption-desorption measurement (Micromeritics TriStar II 3020, Norcross, GA, USA). The morphologies were observed by scanning electron microscopy (SEM, JSM-7900F, JEOL, Tokyo, Japan). The microcrystalline structure and elemental distributions of samples were observed with the field emission transmission electron microscopy (HR-TEM, JEM-2100F, JEOL, Tokyo, Japan) equipped with energy-dispersive X-ray spectroscopy (EDS). The thermogravimetric (TG) analysis was performed by a thermal analyzer (TG-DSC, NETZSCH STA 449F3, NETZSCH, Selb, Germany) under a nitrogen atmosphere with temperature ramp of 5 °C min^-1^. The TGA of Figure S3 was carried out under air atmosphere. The surface chemical species were analyzed by X-ray photoelectron spectra (XPS, Thermo Scientific K-Alpha, Thermo Scientific, Waltham, MA, USA). The elemental composition was analyzed by inductively coupled plasma-mass spectrometry (ICP-MS, Shimadzu ICPE-9000, Shimadzu, Kyoto, Japan). The adsorption ability of cathode materials for LiPSs was measured in Li_2_S_4_ solution. Sublimed sulfur and Li_2_S (with a mole ratio of 3:1) were dispersed in 1, 2-dimethoxyethane (DME) and 1, 3-dioxolane (DOL) (with a volume ratio of 1:1), and then the mixture was stirred at 60 °C for 12 h in an argon-filled sealed bottle. Finally, 0.02 M Li_2_S_4_ solution was obtained. 

### 3.5. Electrochemical Measurements

The working electrodes were prepared by mixing 88 wt% of HPCs/S or HPCs/S@MnO_2_, 3 wt% of Super P, 3 wt% of carbon nanotubes, 2 wt% of carboxymethyl cellulose (CMC), and 4 wt% of styrene butadiene rubber (SBR) in deionized water to form a homogeneous slurry, and then the slurry was pasted on carbon-coated Al foil with a sulfur loading of 4, 7, 10 mg cm^−2^. CR2032 coin type cells were assembled in an Ar-filled glovebox (9555, MBRAUN CHINA, Shanghai, China, H_2_O, O_2_ < 1 ppm) using HPCs/S and HPCs/S@MnO_2_ as the working electrodes, lithium metal as the counter electrode, Celgard 2400 (Celgard, Charlotte, NC, USA) as the separator, and 1 M LiTFSI in DOL/DME (with a volume ratio of 1:1) with the addition of 1 wt% LiNO_3_ as the electrolyte. The galvanostatic discharge/charge tests were conducted by a battery test system (CT2001A, LAND, Wuhan, China) in a voltage window of 1.7–2.8 V. The cyclic voltammetry (CV) and electrochemical impedance spectroscopy (EIS) tests were performed via an electrochemical workstation (Bio-Logic VSP, Grenoble, France).

## 4. Conclusions

In summary, we constructed a HPCs/S@MnO_2_ core-shell structured cathode for Li–S batteries by in situ growth of δ-MnO_2_ nanosheets onto hierarchically porous carbon microsphere/sulfur composites. The inner conductive HPCs synthesized by spray-drying possess a large pore volume and large specific surface area, which can encapsulate a high mass of sulfur and offer fast Li-ion/electron transport channels. The outer MnO_2_ nanosheets can effectively improve the conversion reaction of LiPSs and suppress the shuttle effect via chemical affinity for LiPSs. Owing to the synergistically structural and physical/chemical confinement of the polysulfide, the HPCs/S@MnO_2_ electrode exhibits excellent performance at high areal sulfur loading. The dense HPCs/S@MnO_2_ electrode with high sulfur loading up to 7.0 mg cm^−2^ delivers areal capacity of 4.0 mAh cm^−2^ at 0.1 C and provides stable cycling stability with a low-capacity decay rate of 0.063 % per cycle after 200 cycles at 0.1 C. Even at a high sulfur loading of 10.0 mg cm^−2^, the designed electrodes still deliver high specific capacity of 1077.2 mAh g^−1^ at 0.05 C. The HPCs/S@MnO_2_ materials were up-scaled and a Li–S pouch cell with a capacity of 2.5 Ah was fabricated. A high specific energy of 270.6 Wh kg^−1^ and enhanced cycle performance were observed. This work offers a feasible method to build advanced sulfur electrodes with high areal loading and sheds light on the commercial application in high-performance Li–S batteries.

## Data Availability

Data are contained within the article and Supplementary Materials.

## Supplementary Materials

The following supporting information can be downloaded at: https://www.mdpi.com/article/10.3390/molecules29245881/s1, Figure S1: Raman spectra of HPCs/S@MnO_2_; Figure S2: The pore size distributions of KB, MWCNTs, and HPCs; Figure S3: The TG curves of HPCs/S@MnO_2_ in air; Figure S4: XPS survey spectrum of HPCs@S@MnO_2_; Figure S5: Cyclic voltammetric (CV) curves of HPCs@S@MnO_2_ and HPCs@S; Figure S6: Discharging curves of the HPCs/S@MnO_2_ with mass loading of 10.0 mg cm^−2^ at 0.05 C; Figure S7: Discharging curves of the pouch type cell at 0.02 C; Figure S8: Digital pictures of a Li_2_S_4_ solution after 1 h upon contact with blank, HPCs, and HPCs@MnO_2_. Table S1: Specific Surface Area and Pore Volume of KB, MWCNTs, HPCs, HPCs/S, and HPCs/S@MnO_2_. Table S2: The Mn concentration of HPCs/S@MnO_2_ performed by ICP-MS. Table S3: The performance comparison of HPCs/S@MnO_2_ cathode with other typical sulfur hosts used in lithium–sulfur batteries.
