# Supplementary material for: Hierarchically Porous Carbon Microspheres Coated with MnO2 Nanosheets as the Sulfur Host for High-Loading Lithium–Sulfur Batteries
Source: Molecules. 2024 Dec 13;29(24):5881. doi: 10.3390/molecules29245881 (PMC11676887; doi:10.3390/molecules29245881)
Supplement: Supplementary file 1 [file molecules-29-05881-s001.zip › molecules-3252023-supplementary.pdf]

Supplementary Materials

A hierarchically porous carbon microspheres coated with MnO_2 nanosheets as the sulfur host for high–loading lithium–sulfur batteries

Liqin Dai [1,2], Zonglin Yi [1,2], Lijing Xie [1,2], Fangyuan Su [1,2], Xiaoqian Guo [1,2], Zhenbing Wang [1,2], Jiayao Cheng [1,2] and Chengmeng Chen [1,2,*]

[1] Shanxi Key Laboratory of Carbon Materials, Institute of Coal Chemistry, Chinese Academy of Sciences, Taiyuan 030001, China

[2] CAS Key Laboratory of Carbon Materials, Institute of Coal Chemistry, Chinese Academy of Sciences, Taiyuan 030001, China

* Correspondence: ccm@sxicc.ac.cn

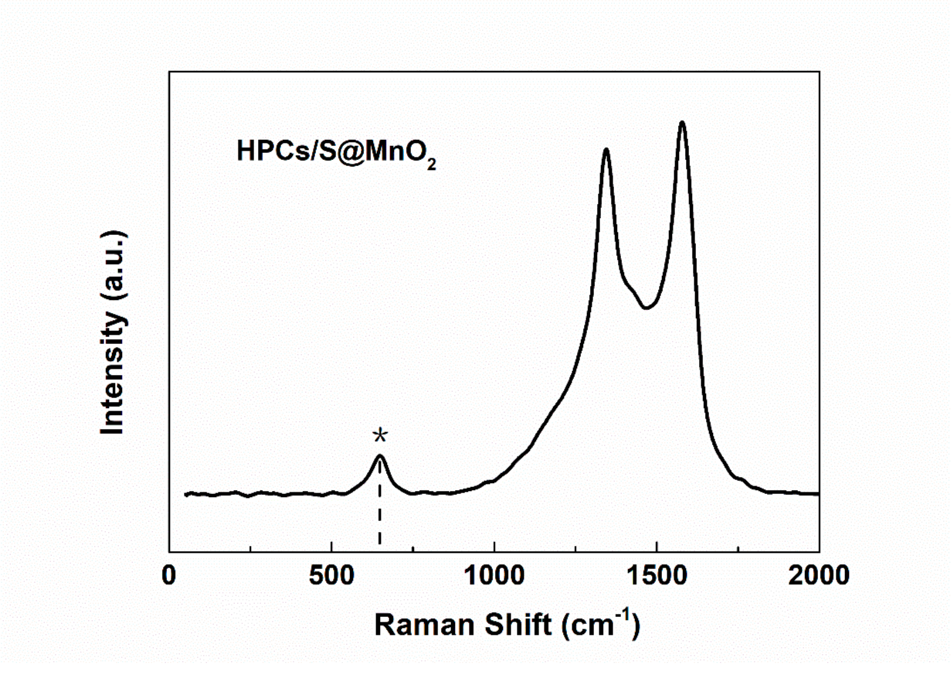

Figure S1. Raman spectra of HPCs/S@MnO_2 (* denotes the characteristic peak of δ–MnO_2)

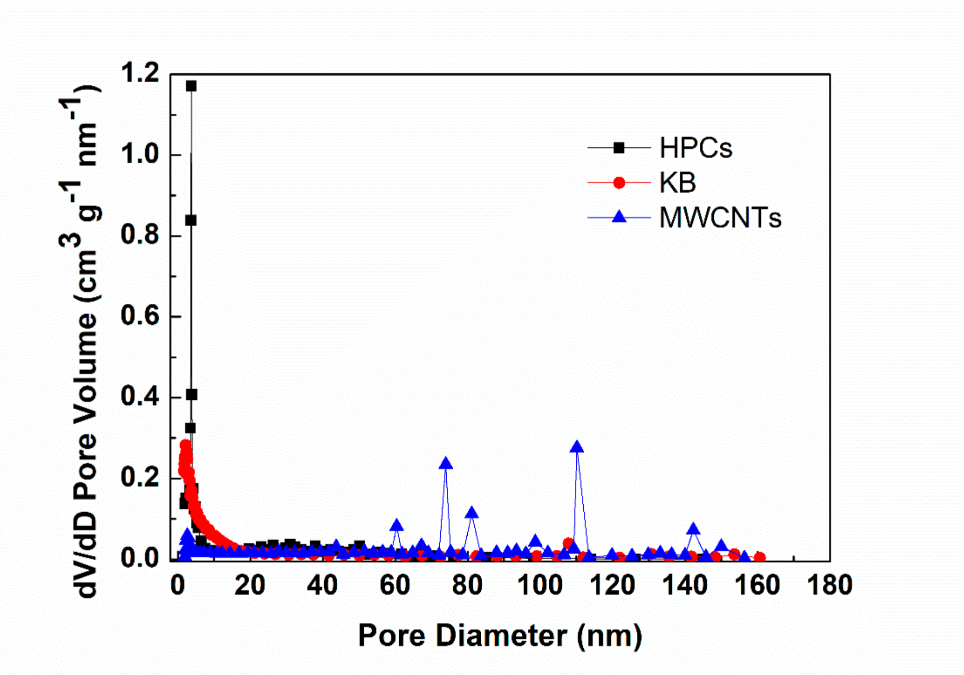

Figure S2. The pore size distributions of KB, MWCNTs and HPCs

Table S1. Specific Surface Area and Pore Volume of KB, MWCNTs, HPCs, HPCs/S and HPCs/S@MnO_2

**Samples**	**S_{BET} m^2 g^{-1}**	**S_{mic} m^2 g^{-1}**	**S_{BJH} m^2 g^{-1}**	**V_{tot} cm^3 g^{-1}**
KB	1301.0	14.7	1286.3	2.590
MWCNTs	305.9	39.0	266.8	2.461
HPCs	957.8	0.2	957.6	2.250
HPCs/S	2.4	0	2.4	0.007
HPCs/S@MnO_2	16.4	1.0	15.4	0.128

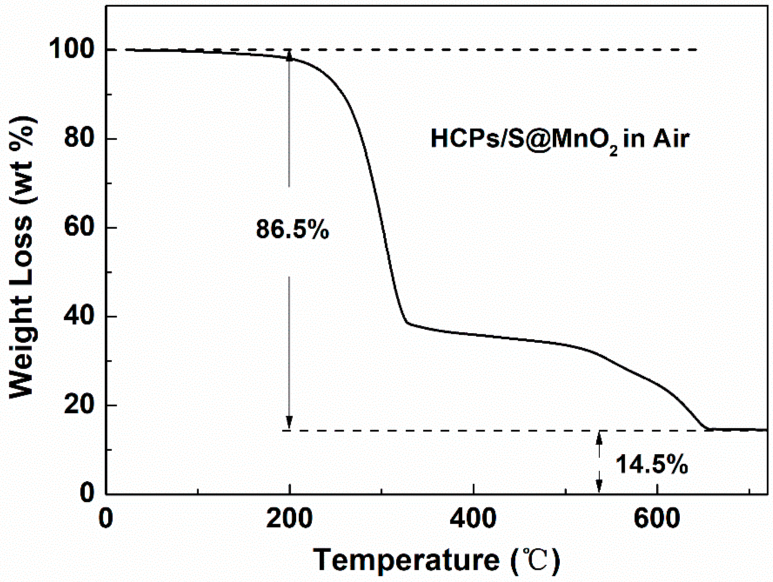

Figure S3. The TG curves of HPCs/S@MnO_2 in air.

Table S2. The Mn concentration of HPCs/S@MnO_2 performed by ICP−MS

Samples	Mn concentration(μg/L)
HPCs/S@MnO_2	139620

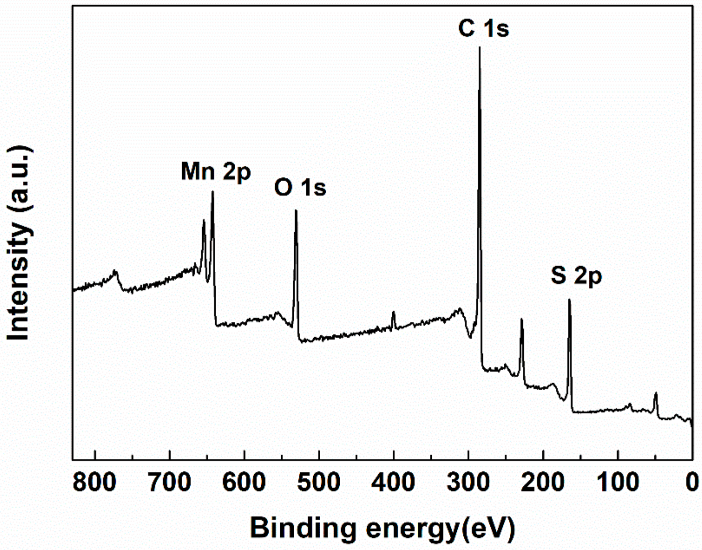

Figure S4. XPS survey spectrum of HPCs@S@MnO_2

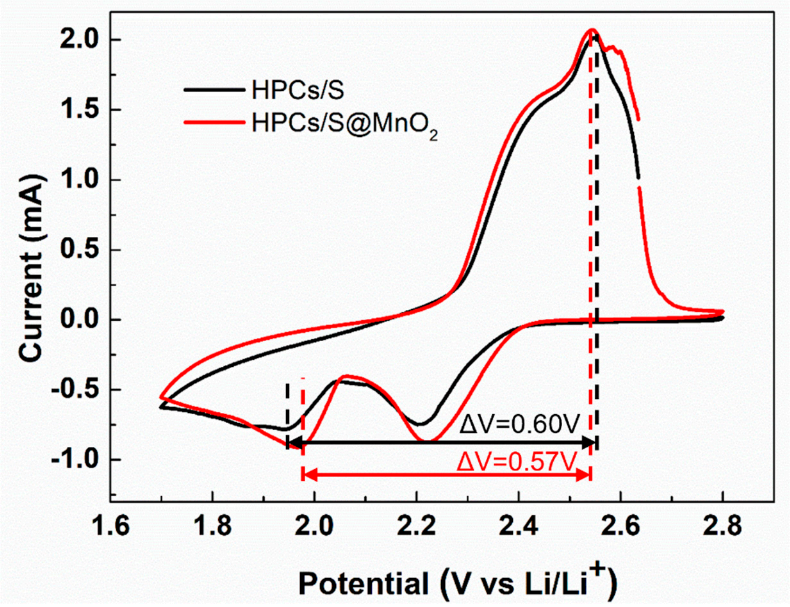

Figure S5. Cyclic voltametric (CV) curves of HPCs@S@MnO_2 and HPCs@S

Table S3. The performance comparison of HPCs/S@MnO_2 cathode with other typical sulfur hosts used in lithium sulfur batteries

Cathode materials	Sulfur loading [mg cm^{-2}]	Cycle number [Current density]	Retention rate [%]	Final discharge Capacity [mAh g^{-1}]	Reference
Lepidocrocite TiO_2/S	2.2	50 [0.3C]	~90.0	~510	50
MoO_2/C/–nano octahedron	2.2	100 [0.2C]	65.8	867	20
S/$NiCo_2O_4$	1.3–1.5	400 [0.5C]	74.1	646	51
S/VO_2-graphene	1.2–1.5	150 [0.5C]	74.0	~670	26
S/V_2O_5-graphene	1.2–1.5	150 [0.5C]	76.0	~710	26
S/N-CNTs/Co-Nanoflowers	6.5	100 [0.2C]	69.0	455	52
S/NCNT@Co-SnS_2	3.0	300 [0.65C]	52.9	562	53
S@MnO_2@polymeric multilayers	0.8	300 [0.1C]	79.9	560	54
S@γ-MnO_2	2.0	300 [0.5C]	85.7	802	17
MnO_2@rGO/S	4.0	200 [0.2C]	60.0	446	55
MnO_2@CF/S	2.6	50 [0.2C]	76.8	~850	56
	4.3	50 [0.2C]	72.7	700	56
**HPCs/S@MnO_2**	**4.0**	**150 [0.1C]**	**95.5**	**607.8**	**This work**
	**7.0**	**200 [0.1C]**	**87.4**	**502.2**	

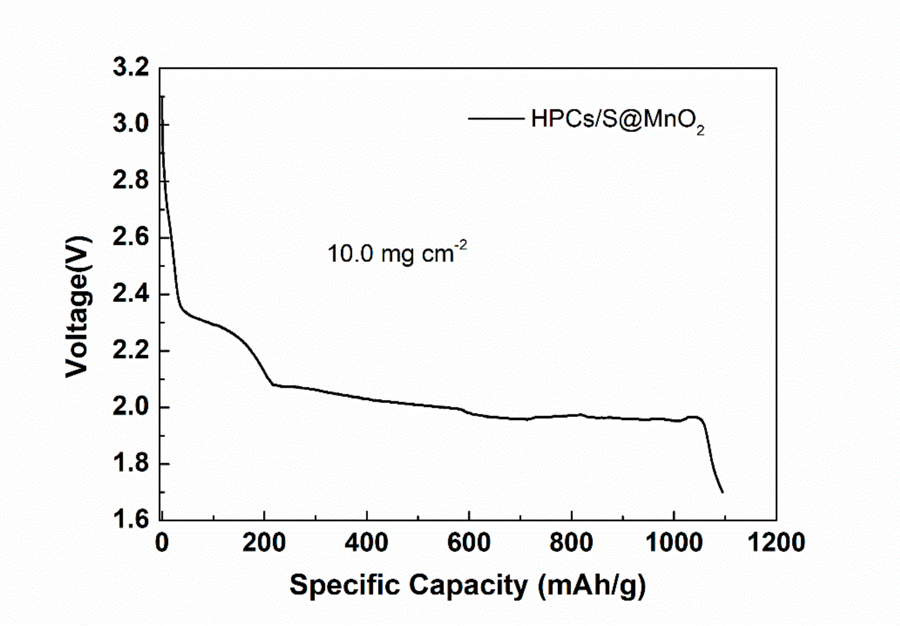

Figure S6. Discharging curves of the HPCs/S@MnO$_2$ with mass loading of 10.0mg cm^{-2} at 0.05C

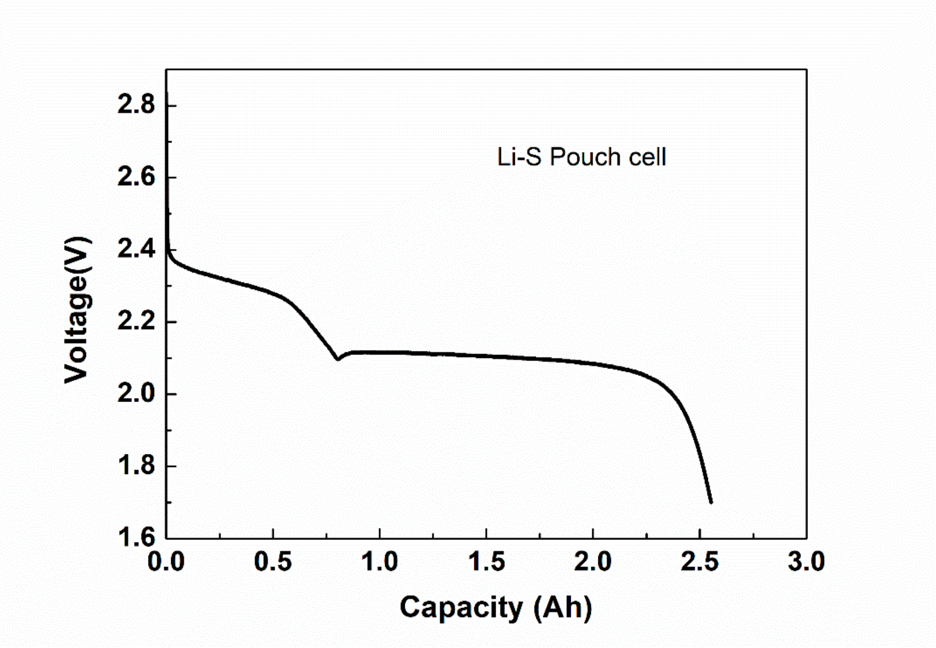

Figure S7. Discharging curves of the pouch−type cell at 0.02 C

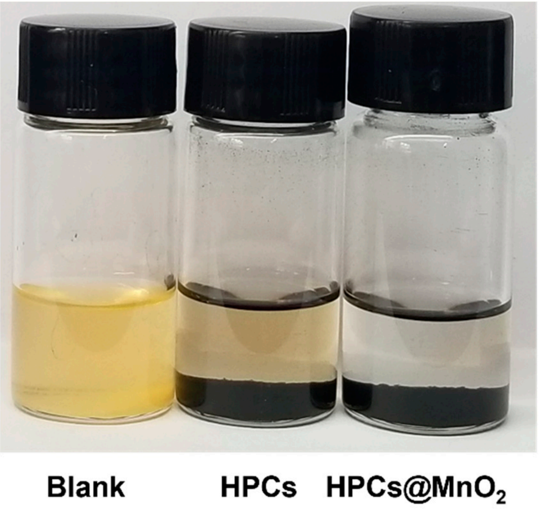

Figure S8. Digital pictures of a Li_2S_4 solution after 1h upon contact with blank, HPCs and HPCs@MnO_2
